# Cholestasis Differentially Affects Liver Connexins

**DOI:** 10.3390/ijms21186534

**Published:** 2020-09-07

**Authors:** Axelle Cooreman, Raf Van Campenhout, Sara Crespo Yanguas, Eva Gijbels, Kaat Leroy, Alanah Pieters, Andrés Tabernilla, Pieter Van Brantegem, Pieter Annaert, Bruno Cogliati, Mathieu Vinken

**Affiliations:** 1 Department of In Vitro Toxicology and Dermato-Cosmetology, Vrije Universiteit Brussel, 1090 Brussels, Belgium; axelle.cooreman@vub.be (A.C.); raf.van.campenhout@vub.be (R.V.C.); screspo@idibell.cat (S.C.Y.); eva.anne.gijbels@vub.be (E.G.); kaat.leroy@vub.be (K.L.); alanah.pieters@vub.be (A.P.); andres.tabernilla.garcia@vub.be (A.T.); 2 Drug Delivery and Disposition, KU Leuven Department of Pharmaceutical and Pharmacological Sciences, 3000 Leuven, Belgium; pieter.vanbrantegem@kuleuven.be (P.V.B.); pieter.annaert@kuleuven.be (P.A.); 3 Department of Pathology, School of Veterinary Medicine and Animal Science, University of São Paulo, São Paulo 05508-270, Brazil; bcogliati@usp.br

**Keywords:** connexin, liver, cholestasis

## Abstract

Connexins are goal keepers of tissue homeostasis, including in the liver. As a result, they are frequently involved in disease. The current study was set up to investigate the effects of cholestatic disease on the production of connexin26, connexin32 and connexin43 in the liver. For this purpose, bile duct ligation, a well-known trigger of cholestatic liver injury, was applied to mice. In parallel, human hepatoma HepaRG cell cultures were exposed to cholestatic drugs and bile acids. Samples from both the in vivo and in vitro settings were subsequently subjected to assessment of mRNA and protein quantities as well as to in situ immunostaining. While the outcome of cholestasis on connexin26 and connexin43 varied among experimental settings, a more generalized repressing effect was seen for connexin32. This has also been observed in many other liver pathologies and could suggest a role for connexin32 as a robust biomarker of liver disease and toxicity.

## 1. Introduction

Gap junctions provide a pathway for the direct exchange of small and hydrophilic molecules and ions between adjacent cells [1,2]. By doing so, they act as goal keepers of the cellular life cycle. In the liver, intercellular communication mediated by gap junctions underlies critical functions, including xenobiotic biotransformation [3,4,5], secretion of albumin [6], glycogenolysis [7], ammonia detoxification [6] and bile secretion [8]. Gap junctions arise from the interaction of two hemichannels of neighboring cells, which in turn are built up by six connexin (Cx) proteins. More than 20 different connexin proteins have been identified in humans and rodents, and all are expressed in a cell type-specific way [9,10]. In the liver, hepatocytes mainly produce Cx32 and small quantities of Cx26, while non-parenchymal liver cells typically harbor Cx43 [11,12,13]. However, connexin expression patterns drastically alter upon liver disease. Our group previously showed that Cx43 production is induced, while Cx32 and Cx26 expression is decreased, in acetaminophen-induced acute liver failure in mice [14]. Likewise, reduced Cx32 and elevated Cx43 levels have been observed in experimental rodent models of liver fibrosis and cirrhosis as well as in human cirrhotic liver [15,16,17,18,19]. However, the expression of these three connexin proteins in cholestasis has been poorly documented thus far. Therefore, the objective of the present study was to investigate whether changes in liver connexin expression, as seen in other liver injuries, can also be detected in cholestatic liver disease.

Cholestasis results from impaired bile secretion with concomitant accumulation of bile acids (BAs) in the liver or systemic circulation [20,21], and can be triggered by a plethora of factors [22]. Depending on the location of the blockage, cholestasis can be classified as extrahepatic or intrahepatic. The clinical manifestation of cholestasis ranges from asymptomatic to symptoms such as fatigue, pruritus and jaundice [23,24]. The bile duct ligation (BDL) model, used in the current study, is a well-known in vivo model to study extrahepatic cholestasis, because the bile flow is obstructed in the extrahepatic bile ducts. In fact, this surgical procedure, typically applied to mice for 20 days, induces different kinds of liver injuries that can be histologically characterized as cholestatic injury, bile ductular proliferation, hepatocellular damage, periportal biliary fibrosis and eventually biliary cirrhosis [25,26,27]. Intrahepatic cholestasis is a consequence of decreased functionality or obstructive lesions of the intrahepatic biliary tract [23]. Depending on the age of the patients, other causes may underlie cholestasis. In children, the driving cause of cholestasis is often linked to the genetic cholestasis syndrome or biliary atresia, while in the adults, cholestasis is more frequently induced by pregnancy, sepsis, biliary obstruction or drugs [24].

Drug-induced cholestasis (DIC) is a specific type of drug-induced liver injury (DILI) [28]. Approximately half of all hepatic drug toxicity cases are associated with DILI [29]. In DILI patients, 20–40% and 12–20% present with cholestatic or mixed cholestatic, and hepatocellular injury, respectively [30]. DIC is of high clinical concern and therefore constitutes a major focus of the current study. Our group recently introduced a new in vitro system to investigate DIC, relying on the treatment of human hepatoma HepaRG cell cultures for 3 days with a concentrated BA mixture and a cholestatic drug, namely atazanavir (ATV), cyclosporin A (CsA) or nefazodone (NEF) [31]. The present study combines this in vitro system with the BDL model, thus providing two experimental settings originating from two different species to study two different types of cholestasis. For both the in vivo and in vitro models, changes in the expression of Cx26, Cx32 and Cx43 were investigated at the transcriptional and translational level using reverse transcription quantitative real-time polymerase chain reaction (RT-qPCR) analysis and semi-quantitative immunoblot analysis, respectively. Furthermore, in situ immunostaining of the three connexin species was performed.

## 2. Results

### 2.1. Effects of Cholestasis on Hepatic Connexin mRNA Expression

**Figure 1:**
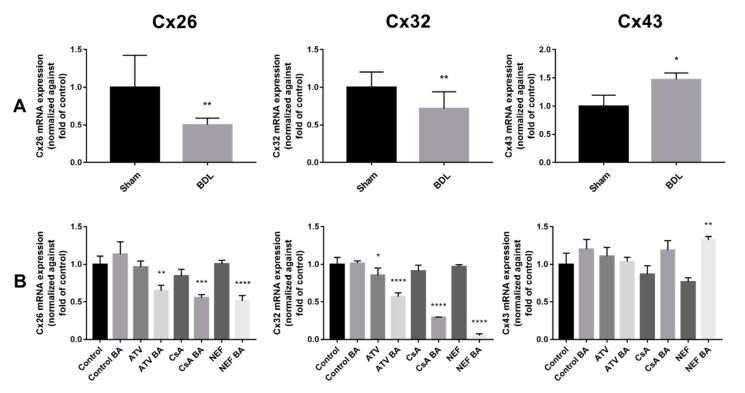
Connexin mRNA expression in cholestasis. Hepatic mRNA levels of Cx26, Cx32 and Cx43 were studied in the liver of cholestatic mice (**A**) and in human hepatoma HepaRG cells cultured in cholestatic conditions (**B**) by RT-qPCR analysis. Relative alterations in mRNA levels were calculated according to the 2^(−ΔΔCq)^ algorithm. (**A**) Liver sections were obtained from male mice following bile duct ligation (BDL) for 20 days. Data were processed by a parametric student t-test with Welch’s correction or a non-parametric Mann–Whitney test. Data are expressed as means +/− SD with * *p* ≤ 0.05 ** *p* ≤ 0.01 compared to sham-operated animals (Sham *n* = 12; BDL *n* = 18) (N = 2). (**B**) Human hepatoma HepaRG cells were exposed to cholestatic drugs either in the absence or presence of a 50× concentrated mixture of bile acids (BA) for 72 h and compared to untreated human hepatoma HepaRG cells, indicated in the figure as control. Data were processed by a parametric one-way ANOVA followed by post hoc tests with Dunnett’s corrections. Data are expressed as means +/− SD with * *p* ≤ 0.05 ** *p* ≤ 0.01 *** *p* ≤ 0.001 and **** *p* ≤ 0.0001 compared to control samples (control *n* = 3; control BA *n* = 3; atazanavir (ATV) *n* = 3; ATV BA *n* = 3; cyclosporine A (CsA) *n* = 3; CsA BA *n* = 3; nefazodone (NEF) *n* = 3; NEF BA *n* = 3) (N = 2).

In the liver, about 90% of the total connexin amount originates from Cx32, while Cx26 and Cx43 each account for 5% of the connexin abundance [32,33]. In several liver diseases, where inflammation and oxidative stress are involved, a switch in mRNA and protein production from Cx32 and Cx26 to Cx43 can be observed [9,34,35]. The upregulation of Cx43 expression is partially due to de novo production by hepatocytes [14]. This effect is also seen in the current study, since hepatic Cx43 mRNA quantities significantly increase, while both Cx26 and Cx32 mRNA amounts decrease following BDL (Figure 1A). The observed downregulation of Cx32 expression could be related to increased degradation of Cx32 mRNA [36]. Similar changes in mRNA patterns are observed for Cx26 and Cx32 in human hepatoma HepaRG cell cultures exposed to cholestatic drugs in the presence of BAs (Figure 1B). Only cells exposed to NEF combined with BAs show a significant increase in Cx43 mRNA expression (Figure 1B).

### 2.2. Effects of Cholestasis on Hepatic Connexin Protein Expression

**Figure 2:**
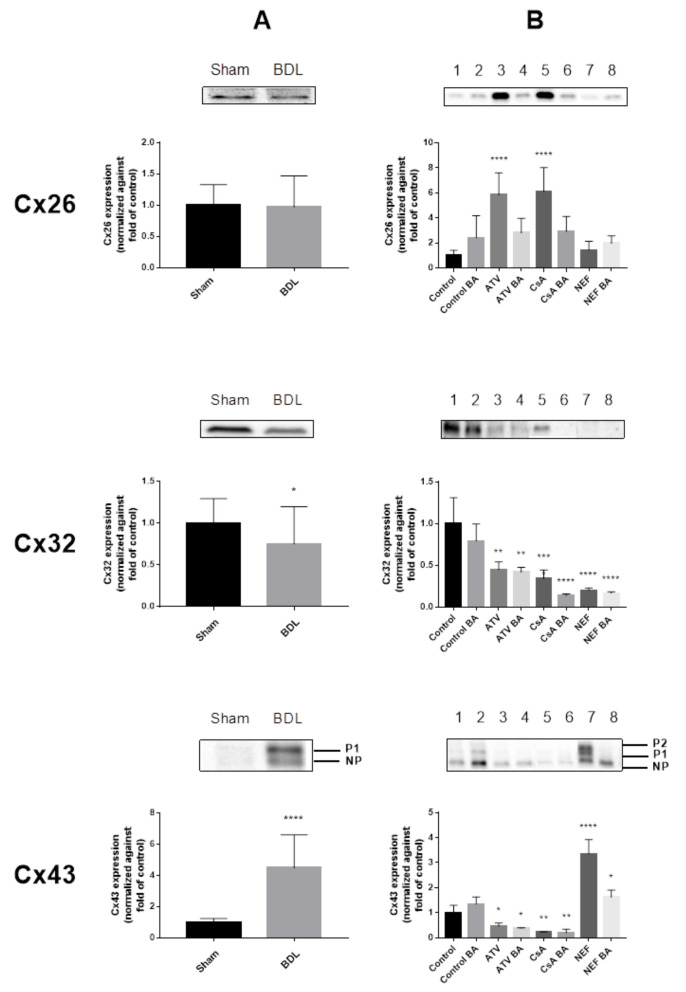
Connexin protein expression in cholestasis. Semi-quantitative immunoblot analysis of Cx26, Cx32 and Cx43 species in livers of cholestatic mice (**A**) and in human hepatoma HepaRG cells cultured in cholestatic conditions (**B**) was performed. For Cx43, both the phosphorylated (P) and non-phosphorylated (NP) variant could be detected. Signals of the three connexins were normalized against total protein loading and expressed as relative alterations compared to sham-operated animals or to control samples, respectively. (**A**) Liver sections were obtained from male mice following bile duct ligation (BDL) for 20 days. Data were processed by a parametric student t-test with Welch’s correction or a non-parametric Mann–Whitney test. Data are expressed as means +/− SD with * *p* ≤ 0.05 and **** *p* ≤ 0.0001 compared to sham-operated animals (Sham *n* = 12; BDL *n* = 18) (N = 1). (**B**) Human hepatoma HepaRG cells were exposed to cholestatic drugs either in the absence or presence of a 50× concentrated mixture of bile acids (BA) for 72 h and compared to untreated human hepatoma HepaRG cells, indicated in the figure as control. The different experimental conditions are presented in the figure as: 1 = control; 2 = control BA; 3 = atazanavir (ATV); 4 = ATV BA; 5 = cyclosporine A (CsA); 6 = CsA BA; 7 = nefazodone (NEF); 8 = NEF BA. Data were processed by a parametric one-way ANOVA followed by post hoc tests with Dunnett’s correction. Data are expressed as means +/− SD with * *p* ≤ 0.05 ** *p* ≤ 0.01 *** *p* ≤ 0.001 and **** *p* ≤ 0.0001 compared to control samples (control *n* = 3; control BA *n* = 3; ATV *n* = 3; ATV BA *n* = 3; CsA *n* = 3; CsA BA *n* = 3; NEF *n* = 3; NEF BA *n* = 3) (N = 1).

**Figure 3:**
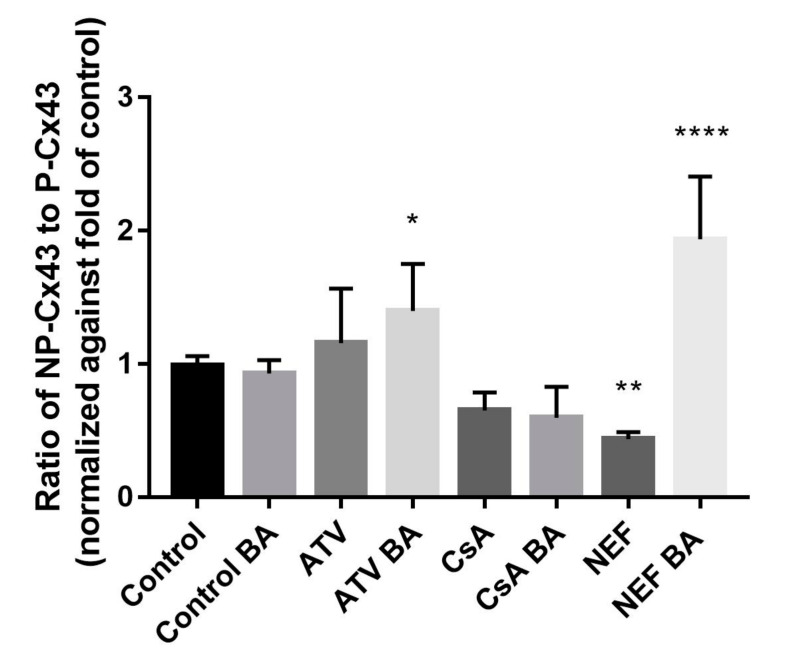
Ratio of protein expression of non-phosphorylated Cx43 to phosphorylated Cx43. Human hepatoma HepaRG cells were exposed to cholestatic drugs either in the absence or presence of a 50× concentrated mixture of bile acids (BA) for 72 h and compared to untreated cells, indicated in the figure as control. Following immunoblot analysis of total Cx43, a second analysis of the expression level of the phosphorylated (P1/P2) and non-phosphorylated (NP) isoforms was performed. The ratio of NP-Cx43 to P1/P2-Cx43 in all different experimental conditions (i.e., 1 = control; 2 = control BA; 3 = atazanavir (ATV); 4 = ATV BA; 5 = cyclosporine A (CsA); 6 = CsA BA; 7 = nefazodone (NEF); 8 = NEF BA) was normalized against total protein loading and expressed as relative alterations compared to control samples. Data were processed by a parametric one-way ANOVA followed by post hoc tests with Dunnett’s correction. Data are expressed as means +/− SD with * *p* ≤ 0.05 ** *p* ≤ 0.01 and **** *p* ≤ 0.0001 compared to control samples (control *n* = 3; control BA *n* = 3; ATV *n* = 3; ATV BA *n* = 3; CsA *n* = 3; CsA BA *n* = 3; NEF *n* = 3; NEF BA *n* = 3) (N = 1).

**Figure 4:**
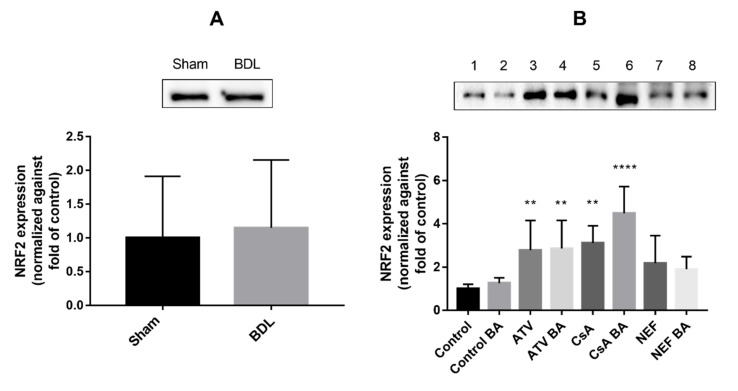
Nrf2 protein expression in cholestasis. Semi-quantitative immunoblot analysis of Nrf2 protein in livers of cholestatic mice (**A**) and in human hepatoma HepaRG cells cultured in cholestatic conditions (**B**) was performed. Signals of Nrf2 were normalized against total protein loading and expressed as relative alterations compared to sham-operated animals or to control samples, respectively. (**A**) Liver sections were obtained from male mice following bile duct ligation (BDL) for 20 days. Data were processed by a parametric student t-test with Welch’s correction. Data are expressed as means +/− SD compared to sham-operated animals (Sham *n* = 12; BDL *n* = 18) (N = 1). (**B**) Human hepatoma HepaRG cells were exposed to cholestatic drugs either in the absence or presence of a 50× concentrated mixture of bile acids (BA) for 72 h and compared to untreated human hepatoma HepaRG cells, indicated in the figure as control. The different experimental conditions are presented in the figure as: 1 = control; 2 = control BA; 3 = atazanavir (ATV); 4 = ATV BA; 5 = cyclosporine A (CsA); 6 = CsA BA; 7 = nefazodone (NEF); 8 = NEF BA. Data were processed by a parametric one-way ANOVA followed by post hoc tests with Dunnett’s correction. Data are expressed as means +/− SD with ** *p* ≤ 0.01 and **** *p* ≤ 0.0001 compared to control samples (control *n* = 3; control BA *n* = 3; ATV *n* = 3; ATV BA *n* = 3; CsA *n* = 3; CsA BA *n* = 3; NEF *n* = 3; NEF BA *n* = 3) (N = 1).

Protein moieties of Cx32 are downregulated in cholestasis both in vivo and in vitro (Figure 2). In human hepatoma HepaRG cell cultures, this effect is amplified by the presence of BAs. Protein levels of Cx26 are unaffected in the mouse BDL model (Figure 2A) and are even elevated in human hepatoma HepaRG cell cultures exposed to ATV or CsA (Figure 2B). These results are in striking contrast to the results obtained by the RT-qPCR analysis in vivo and in vitro, in which mRNA quantities of Cx26 were decreased (Figure 1). Increased protein expression of hepatic Cx26 has been suggested to reflect a compensatory response to the downregulation of Cx32 in an inflammatory environment [37]. For Cx43, the results of the immunoblot analysis are less consistent. Thus, while there is an increase in Cx43 production in the liver of cholestatic mice and in human hepatoma HepaRG cell cultures treated with NEF, Cx43 protein levels are negatively affected by both ATV and CsA in vitro. This could suggest a drug-specific effect. Posttranslational phosphorylation can be detected for Cx43 via immunoblot analysis. Typically, three bands appear for Cx43 at different molecular weights depending on the migration rate of the corresponding isoforms. These isoforms include the lowest or fast-migrating non-phosphorylated Cx43 (NP-Cx43) and two slow-migrating phosphorylated isoforms of Cx43 (P1-Cx43 and P2-Cx43) [38]. While P1-Cx43 and P2-Cx43 can be distinguished in human hepatoma HepaRG cell cultures, only one phosphorylated Cx43 variant can be detected in mouse liver (Figure 2). The ratio of the expression level of NP-Cx43 to the expression level of P1/P2-Cx43 was determined for both the in vivo and in vitro setting. For the former, no differences were observed between sham-operated mice and mice subjected to BDL, and NP-Cx43 and P1/P2-Cx43 levels were proportionally elevated (data not shown). Human hepatoma HepaRG cell cultures only exposed to NEF showed a higher level of the phosphorylated Cx43 isoforms (Figure 3). On the other hand, the upregulated Cx43 expression levels in human hepatoma HepaRG cell cultures exposed to NEF in the presence of BAs could be attributed to an increase in NP-Cx43. A shift in expression from the phosphorylated isoforms, localized in the cell plasma membrane, to the non-phosphorylated isoform, localized in the cytoplasm, has been reported to occur in cancer [35,39,40,41]. Moreover, Cx43 predominantly appears in its non-phosphorylated variant in the liver [35,41]. Changes in connexin expression in liver disease have been linked to several processes, such as oxidative stress [35]. Therefore, the production of nuclear factor erythroid 2-related factor 2 (Nrf2), a transcription factor counteracting cholestasis by regulating anti-oxidative genes, was assessed by immunoblot analysis in both the in vivo and in vitro models (Figure 4). Human hepatoma HepaRG cells treated with ATV and CsA with and without BAs showed a steep increase in Nrf2 expression (Figure 4B). Both NEF and BDL tended to increase Nrf2 expression, yet these effects were not significant (Figure 4A,B).

**Figure 5:**
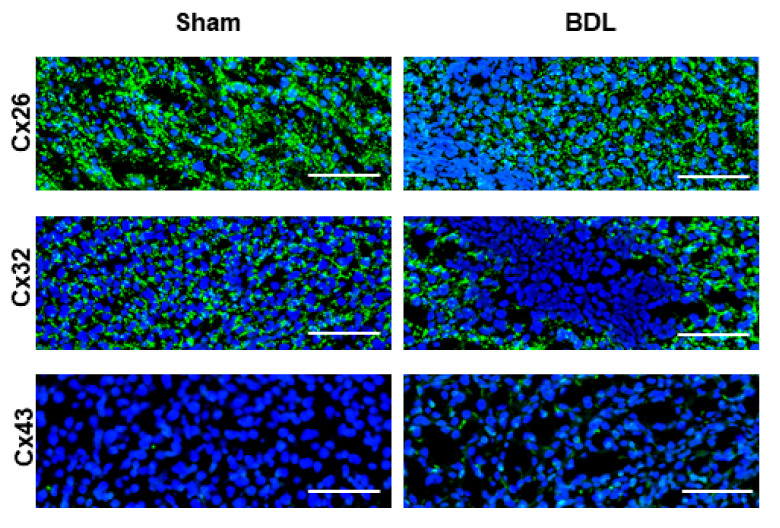
Connexin protein localization in livers of cholestatic mice. Liver sections were obtained from male mice following bile duct ligation (BDL) for 20 days. Cellular localization of Cx26, Cx32 and Cx43 (green) was revealed by immunohistochemistry analysis with nuclear counterstaining using DAPI (blue). Scale bar, 100 µm (Sham *n* = 3; BDL *n* = 3) (N = 1).

**Figure 6:**
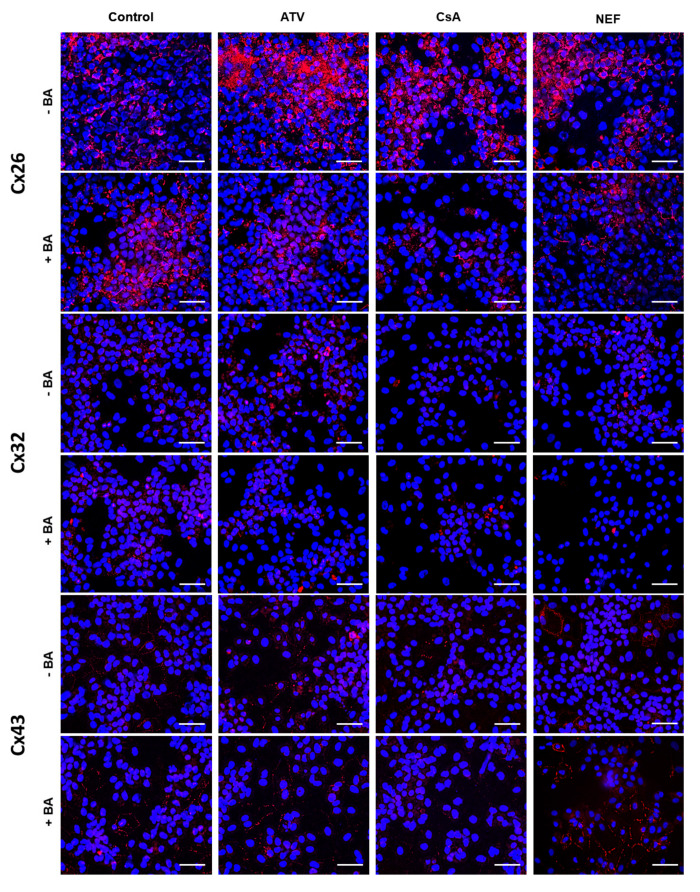
Connexin protein localization in human hepatoma HepaRG cells cultured in cholestatic conditions. Human hepatoma HepaRG cells were exposed to cholestatic drugs either in the absence or presence of a 50× concentrated mixture of bile acids (BA) for 72 h and compared to untreated human hepatoma HepaRG cells, indicated in the figure as control. Cellular localization of the three connexin species, namely Cx26, Cx32 and Cx43 (red), was revealed by immunocytochemistry analysis with nuclear counterstaining using DAPI (blue). Scale bar, 50 µm; samples (control *n* = 3; control BA *n* = 3; ATV *n* = 3; ATV BA *n* = 3; CsA *n* = 3; CsA BA *n* = 3; NEF *n* = 3; NEF BA *n* = 3) (N = 1).

**Figure 7:**
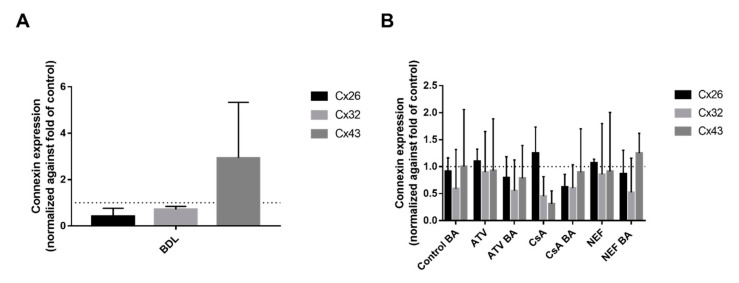
Quantification of immunohistochemistry and immunocytochemistry analysis results. Cellular localization of Cx26, Cx32 and Cx43 was revealed by immunohistochemistry (Figure 5) or immunocytochemistry (Figure 6) analysis with nuclear counterstaining using DAPI, followed by quantification of the obtained images via ImageJ software. The ratio of the area of occurrence of the particular connexin to the number of nuclei was measured and expressed as relative alterations compared to the control. (**A**) Liver sections were obtained from male mice following bile duct ligation (BDL) for 20 days. Data were processed by a parametric student t-test with Welch’s correction. Data are expressed as means +/− SD compared to sham-operated animals (Sham *n* = 3; BDL *n* = 3) (N = 1). (**B**) Human hepatoma HepaRG cells were exposed to cholestatic drugs either in the absence or presence of a 50× concentrated mixture of bile acids (BA) for 72 h and compared to untreated human hepatoma HepaRG cells, indicated in the figure as control. Data were processed by a parametric one-way ANOVA followed by post hoc tests with Dunnett’s correction. Data are expressed as means +/− SD compared to control samples (control *n* = 3; control BA *n* = 3; ATV *n* = 3; ATV BA *n* = 3; CsA *n* = 3; CsA BA *n* = 3; NEF *n* = 3; NEF BA *n* = 3) (N = 1).

In normal liver, both Cx32 and Cx43 are evenly distributed in different acinar areas, whereas Cx26 is preferentially expressed by periportal hepatocytes [11,42,43]. Although mainly located at the cell plasma membrane, a considerable portion of the connexin population resides in the cytoplasm of cells, which may reflect the rapid turnover of these proteins in vitro [44] and in vivo [45]. In hepatocytes, gap junctions occupy about 3% of the cell plasma membrane area [46]. This appears as a dotted pattern upon immunostaining, which was also seen in the present study for both Cx26 and Cx32 in the liver of sham-operated mice (Figure 5) and in the untreated human hepatoma HepaRG cells (Figure 6). The appearance of Cx32, and to a lesser extent, Cx26, was decreased in mice subjected to BDL in line with the immunoblot analysis (Figure 2A). In human hepatoma HepaRG cell cultures exposed to cholestatic drugs, whether together with BAs or not, the presence of Cx32 was reduced (Figure 6). A trend towards increased expression of Cx26 was seen upon immunocytochemistry analysis of human hepatoma HepaRG cells exposed to ATV and CsA, which is in agreement with the results of the protein expression analysis (Figure 2B). By contrast, when human hepatoma HepaRG cells were exposed to the cholestatic drugs together with BAs, Cx26 immunosignals tended to decrease. Opposite observations applied to Cx43, which became increasingly expressed upon BDL, an effect also seen in human hepatoma HepaRG cells exposed to NEF, mainly in combination with BAs (Figure 6). As in the immunoblot analysis, decreased Cx43 presence was observed in human hepatoma HepaRG cells exposed to CsA with and without BAs. Although the results of the immunoblot analysis showed lowered expression of Cx43 in a cholestatic environment induced by ATV, this effect, and the effects on the other connexin species, could not be quantitatively confirmed by the immunocytochemistry analysis (Figure 7).

## 3. Discussion

Several groups have described loss of Cx26 and, more prominently, of Cx32, with concomitant upregulation of Cx43 production in various liver diseases, both in experimental animals and in clinical patients [9,34]. Almost three decades ago, decreased Cx32 in rat liver following BDL was described [47]. This was later confirmed using a rodent model of acute-on-chronic liver failure, which also showed downregulated Cx32 and Cx26 production, but elevated Cx43 expression [48]. This complies with the results of the mouse model of cholestatic liver injury addressed in the present study, albeit with the decrease in Cx26 production mainly restricted to the transcriptional level. Thus, while downregulation of Cx32 production may be the result of both altered protein turnover and transcriptional mechanisms, reduction of Cx26 expression seems primarily regulated by the mRNA machinery. In this respect, oxidative stress and inflammation, which accompany cholestatic insults, have been repeatedly shown to negatively affect Cx26 and Cx32 protein and/or mRNA expression in the liver [36,49,50,51,52]. Deterioration of Cx32 during inflammatory conditions in the liver results from mRNA degradation [53]. Protein and mRNA levels of Cx32 were decreased in the in vitro model of cholestasis in all cholestatic conditions. Reduced Cx32 expression in cholestasis, both in vitro and in vivo, could possibly confirm the cytoprotective role previously assigned to Cx32. Indeed, in several chronic liver injury types, downregulation of Cx32 production has been associated with increased liver damage, inflammation and oxidative stress [54,55]. Cx32 mRNA levels significantly decreased only in human hepatoma HepaRG cell cultures exposed to cholestatic drugs together with BAs. The presence of the concentrated mix of BAs in the in vitro system creates an environment more comparable to the in vivo situation, since cholestasis patients present with 30–50× increased concentrations of serum BA [21,56,57]. The addition of BAs seems to potentiate the effect of cholestatic drugs [31]. The sensitizing effect of BAs was also observed for Cx26, as mRNA levels were reduced following exposure of the human hepatoma HepaRG cells to cholestatic drugs and BAs. This was not mirrored at the translational level, where an increase in Cx26 protein was seen for ATV and CsA, possibly as a compensatory response to the downregulation of Cx32 protein production [37]. Unlike Cx26 and Cx32, Cx43 production tended to increase in mouse liver following cholestasis induction. This could, however, not be reproduced in vitro. The discrepancy between the in vivo and in vitro results could have various reasons. While the BDL animal model triggers extrahepatic cholestasis, the DIC in vitro system recapitulates intrahepatic cholestasis. Besides the nature of the cholestasis response, there might also be an interspecies difference involved. BA composition and individual BA concentrations in rodents and humans are considerably distinct from each other [58,59,60]. Furthermore, dissimilarities in cell types could play a role. In this respect, human hepatoma HepaRG cell cultures consist of hepatocyte-like cells and cholangiocyte-like cells, while liver tissue, in casu of murine origin, also contains non-parenchymal cells, including Kupffer cells, hepatic stellate cells, liver sinusoidal endothelial cells and lymphocytes [61,62]. Our group previously demonstrated that the increase in Cx43 production in the liver following acetaminophen overdosing is due to both upregulated expression of Cx43 by non-parenchymal liver cells as well as de novo production by hepatocytes [14]. Elevated Cx43 levels may also reflect migration of oval cells into damaged areas. These stem cell-like progenitor cells can differentiate into hepatocytes or biliary epithelial cells and express Cx43 in early phases of proliferation [63,64,65,66]. BDL is known to induce proliferation of oval cells, the so-called ductular reaction [27,67], which could explain the abundant presence of Cx43 in the livers of cholestatic mice. Cx43 gene expression is controlled by the transcription factor activator protein-1, which is composed of the proto-oncogenes *c*-fos and *c*-jun [68]. In rat myometrium, activator protein-1 activates Cx43 expression in stress conditions [69]. A similar scenario may take place in the liver upon cholestasis. This is substantiated by the acknowledged induced expression of *c*-fos and *c*-jun in rodent liver triggered by BDL [70]. While Cx43 expression was elevated both at the transcriptional and translational level in the mouse BDL model, such an effect was only seen for NEF in human hepatoma HepaRG cell cultures. Moreover, a switch from the phosphorylated isoforms to the non-phosphorylated Cx43 variant was observed when cells were exposed to NEF in combination with BAs. In general, the outcome of cholestasis on Cx43 in vitro seems to depend on the nature of the drug. In this light, three triggering factors of DIC have been identified, namely transporter changes, hepatocellular changes and altered bile canaliculi dynamics [31], where all are differentially affected by ATV, CsA and NEF. These triggering factors result in BA accumulation, which in turn induces two cellular responses. The deteriorative response is typified by the occurrence of mitochondrial impairment and inflammation resulting in oxidative stress, which in turn leads to endoplasmic reticulum stress. Nrf2, a regulator of anti-oxidative responses by enhancing the expression of anti-oxidative and cytoprotective proteins, was increasingly expressed in HepaRG cells treated with ATV and CsA. This confirms the activation of the deteriorative response and complies with results previously obtained by our group using transcriptomics analysis [31]. Despite the fact that an increase in Nrf2 expression was not observed upon BDL, the hepatoprotective role of Nrf2 in cholestasis was shown in other studies [71,72]. The second cellular response, the adaptive cellular response, attempts to counteract the deteriorative response by activation of a number of nuclear receptors [21]. Our group recently reported divergent transcriptomic profiles induced by ATV, CsA and NEF in vitro, which could be related to the different effects on connexins observed in the present study [31].

In conclusion, our results show that cholestasis affects connexin expression both in vivo and in vitro. While the changes in the expression patterns of Cx26 and Cx43 varied among experimental settings (i.e., in vivo or in vitro) or, for the in vitro model, among cholestatic drugs (i.e., ATV, CsA or NEF), a more generalized response was seen for Cx32. Cx32 production was downregulated at both levels (i.e., transcriptional and translational) in liver samples of mice subjected to BDL as well as in samples from human hepatoma HepaRG cell cultures exposed to cholestatic drugs (i.e., ATV, CsA and NEF) in the presence of BAs. This has also been observed in many other liver pathologies [9] and could suggest a role for Cx32 as a robust biomarker of liver disease and toxicity.

## 4. Materials and Methods

### 4.1. Animals and Treatment

Male C57BL/6 mice were obtained from Jackson Laboratories (USA). Animals were housed in the animal facility of the School of Veterinary Medicine and Animal Science of the University of São Paulo (FMVZ-USP) in Brazil. Mice were kept in a room with ventilation (i.e., 16–18 air changes/h), relative humidity (i.e., 45–65%), controlled temperature (i.e., 20–24 °C) and light/dark cycle 12:12, and were given water and balanced diet (NUVILAB-CR1, Nuvital Nutrientes LTDA, Brazil) ad libitum. This study was approved by the Committee on Bioethics of FMVZ-USP (protocol number 9999100314) and all animals received humane care according to the criteria outlined in the “Guide for the Care and Use of Laboratory Animals”. The surgical BDL and sham procedures, considered as control, were set up in 8-week to 12-week-old mice as described elsewhere [25,73]. Mice were sacrificed 20 days after BDL by exsanguination during sampling under isoflurane-induced anesthesia. Blood collected by cardiac puncture was drawn into a heparinized syringe and centrifuged for 10 min at 1503× *g*, and serum was stored at −20 °C. Livers were excised and fragments were fixed in 10% phosphate-buffered formalin or snap-frozen in liquid nitrogen with storage at −80 °C. This model has been previously characterized by our group and defined as cholestatic and fibrotic based on morphometric analysis of liver collagen, and spectrophotometric determination of serum levels of alanine aminotransferase, aspartate aminotransferase, alkaline phosphatase and conjugated and total bilirubin [74].

### 4.2. Cell Cultures and Treatment

Cell cultures were set up as recently described by our group [31]. In essence, 24-well plates were coated with a 0.1 mg/mL collagen solution consisting of collagen type I (Corning, United Kingdom) diluted in 0.02 N acetic acid (Sigma-Aldrich, Overijse, Belgium). The collagen solution was subsequently removed and the cell culture plates were washed with phosphate-buffered saline (PBS). Cryopreserved differentiated human hepatoma HepaRG cells (Biopredic International, Saint-Grégoire, France) were thawed and seeded following the manufacturer’s instructions with basal hepatic cell culture medium (i.e., Williams’ E basal medium with GlutaMAX containing phenol red (MIL600C, Biopredic International, Saint-Grégoire, France)) supplemented with thaw seed and general purpose cell culture medium (ADD670C, Biopredic International, France). The cells were seeded at a density of 0.48 × 10^6^ cells/well in 500 μL/well of the cell culture medium. Cell culture medium was changed every 2–3 days with basal hepatic cell culture medium supplemented with maintenance and metabolism cell culture medium (ADD620C, Biopredic International, Saint-Grégoire, France). Stock solutions of ATV (Sigma-Aldrich, Overijse, Belgium), CsA (Calbiochem, San Diego, CA, USA) and NEF (Sigma-Aldrich, Overijse, Belgium) at 60 mM, 20 mM and 30 mM, respectively, were prepared in dimethyl sulfoxide (DMSO) (Sigma-Aldrich, Overijse, Belgium). The concentrated stock solutions were diluted 1000× ex tempore in basal hepatic cell culture medium supplemented with induction serum-free cell culture medium (ADD650C, Biopredic International, Saint-Grégoire, France). All experimental conditions contained a final DMSO concentration of 0.25% v/v. A 50× concentrated mixture of five BAs (i.e., 66 µM glycochenodeoxycholic acid, 20 µM deoxycholic acid, 19.5 µM chenodeoxycholic acid, 19 µM glycodeoxycholic acid, and 17.5 µM glycocholic acid (Sigma-Aldrich, Overijse, Belgium)) was prepared [75]. As such, four experimental conditions were implemented, namely (i) untreated human hepatoma HepaRG cells, (ii) human hepatoma HepaRG cells treated with 50× concentrated BA mix, (iii) human hepatoma HepaRG cells treated with a cholestatic drug (i.e., ATV, CsA or NEF), and (iv) human hepatoma HepaRG cells treated with 50× concentrated BA mix and a cholestatic drug (i.e., ATV, CsA or NEF). All conditions were applied for 72 h with cell culture medium being renewed every 24 h.

### 4.3. Reverse Transcription Quantitative Real-Time Polymerase Chain Reaction Analysis

Total RNA was extracted from mouse liver tissue or from human hepatoma HepaRG cells using a GenEluteTM Mammalian Total RNA purification Miniprep Kit (Sigma-Aldrich, Overijse, Belgium) and the On-column DNase I digestion Set (Sigma-Aldrich, Overijse, Belgium) according to the manufacturer’s instructions. The isolated RNA was spectrophotometrically measured using a NanoDrop^®^ 2000 Spectrophotometer (Thermo Fisher Scientific, Waltham, MA, USA) to assess purity and quantity. A cut-off ratio between 1.8 and 2.1 for the absorption at 260/280 nm was used for assessing purity. Next, the synthesis and amplification of cDNA as well as the RT-qPCR analysis were performed as explained elsewhere [76]. TaqMan probes and primers specific for the target and reference genes are depicted in Table 1. Relative alterations (fold change) in mRNA levels were calculated according to the 2^(−ΔΔCq)^ algorithm [77].

### 4.4. Immunoblot Analysis

**Figure 8:**
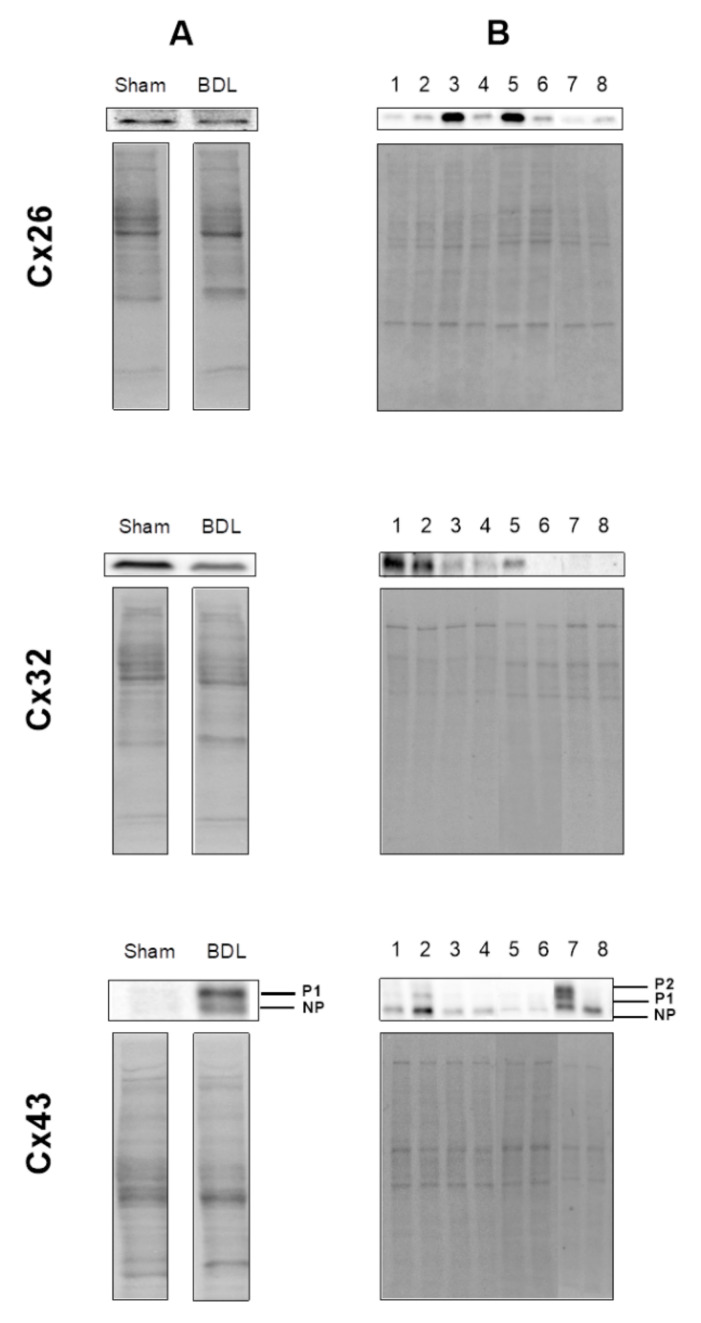
Total protein loading of samples used in immunoblot analysis. Semi-quantitative immunoblot analysis of Cx26, Cx32 and Cx43 in liver of cholestatic mice (**A**) and in human hepatoma HepaRG cells cultured in cholestatic conditions (**B**) was performed. For Cx43, both the phosphorylated (P) and non-phosphorylated (NP) variant could be detected. Signals of the three connexins were normalized against total protein loading, which are shown for two representative samples. (**A**) Liver sections were obtained from male mice following bile duct ligation (BDL) for 20 days (Sham *n* = 12; BDL *n* = 18) (N = 1). (**B**) Human hepatoma HepaRG cells were exposed to cholestatic drugs either in the absence or presence of a 50× concentrated mixture of bile acids (BA) for 72 h and compared to untreated human hepatoma HepaRG cells, indicated in the figure as control. The different experimental conditions are presented in the figure as: 1 = control (*n* = 3); 2 = control BA (*n* = 3); 3 = atazanavir (ATV) (*n* = 3); 4 = ATV BA (*n* = 3); 5 = cyclosporine A (CsA) (*n* = 3); 6 = CsA BA (*n* = 3); 7 = nefazodone (NEF) (*n* = 3); 8 = NEF BA) (*n* = 3) (N = 1).

Immunoblot analysis of mouse liver tissue and human hepatoma HepaRG cells was performed as previously described [38]. Briefly, liver tissue was weighed, and for each mg of liver tissue, 10 µL of lysis buffer was added. While keeping them on ice, the samples were homogenized using a mixer. Human hepatoma HepaRG cells were washed, scraped and collected in the presence of ice-cold PBS. Following centrifugation, cell pellets were resuspended in lysis buffer and sonicated for 30 s with 50% pulse while keeping the cells on ice. After shaking the in vivo and in vitro samples for 15 min on a rotator at 4 °C, the samples were centrifuged at 14,000× *g* for 15 min at 4 °C. Finally, the supernatant of each sample was transferred to a new tube and the amount of protein was quantified by means of a bicinchoninic assay. Following electrophoresis and blotting, nitrocellulose or polyvinylidene fluoride membranes were incubated with 5% non-fatty milk (Régilait, Saint-Martin-Belle-Roche, France) in Tris-buffered saline solution (i.e., 20 mM Tris and 135 mM sodium chloride) containing 0.1% Tween 20 (Sigma-Aldrich, Overijse, Belgium). Membranes were incubated overnight at 4 °C with primary antibody directed against Cx26 (51-2800, Thermo Fisher Scientific, Waltham, MA, USA), Cx32 (C3470, Sigma-Aldrich, Overijse, Belgium), Cx43 (C6219, Sigma-Aldrich, Overijse, Belgium) and Nrf2 (16396-1-AP, Proteintech, Manchester, United Kingdom), followed by incubation for 1 h at room temperature with polyclonal goat anti-rabbit secondary antibody (Dako, Santa Clara, CA, USA) (Table 2). Detection of the proteins was carried out using enhanced chemiluminescence. For semi-quantification purposes, a normalization method based on total protein loading was used to overcome the drawbacks associated with the use of housekeeping proteins [78]. Accordingly, Cx26, Cx32, Cx43 and Nrf2 signals in mouse liver tissue and human hepatoma HepaRG samples were normalized against total protein loading and expressed as relative alterations compared to sham-operated animals and untreated human hepatoma HepaRG cells, respectively, considered as control (Figure 8).

### 4.5. Immunohistochemistry Analysis

Immunohistochemistry analysis was performed as previously described [79] with slight modifications. Flash frozen mouse liver tissue samples were embedded in Tissue Freezing Medium^®^ (Leica, Wetzlar, Germany). Then, 10 µm thick liver sections were fixed in acetone for 10 min at −20 °C. Liver sections were incubated with primary antibodies directed against Cx26 (51-2800, Thermo Fisher Scientific, Waltham, MA, USA), Cx32 (C3470 Sigma-Aldrich, Overijse, Belgium) and Cx43 (C6219, Sigma-Aldrich, Overijse, Belgium) in blocking buffer containing 5% donkey serum (Jackson Immunoresearch Inc., West Grove, PA, USA) and 1% bovine serum albumin (Sigma-Aldrich, Overijse, Belgium) for 1 h at 37 °C (Table 2). After extensive rinsing with PBS supplemented with 0.5% Tween-20 (Sigma-Aldrich, Overijse, Belgium), samples were incubated with appropriate Alexa Fluor^®^ 488-conjugated secondary antibody (Jackson Immunoresearch Inc., West Grove, PA, USA). Nuclei were stained with 4′,6-diamidino-2-phenylindole (DAPI) and samples were mounted with Vectashield (Vector Laboratories, Burlingame, CA, USA). Detection was performed by fluorescence microscopy (20× objective) (Nikon Eclipse Ti, Tokyo, Japan). Images were processed and quantified with ImageJ software (version 1.52, USA). The ratio of the area of occurrence of the particular connexin to the number of nuclei was measured and expressed as relative alterations compared to sham-operated animals, considered as control.

### 4.6. Immunocytochemistry Analysis

Human hepatoma HepaRG cells were washed with ice-cold PBS and fixed by incubating with a mixture of equal amounts of ethanol and acetone for 10 min at −20 °C (Sigma-Aldrich, Overijse, Belgium). Following rinsing, cells were blocked with blocking buffer containing 5% donkey serum (Jackson Immunoresearch Inc., West Grove, PA, USA) and 1% bovine serum albumin (Sigma-Aldrich, Overijse, Belgium) for 45 min at room temperature. Cells were incubated overnight at 4 °C with primary antibodies directed against Cx26 (51-2800, Thermo Fisher Scientific, Waltham, MA, USA), Cx32 (3470, Sigma-Aldrich, Overijse, Belgium) and Cx43 (C6219, Sigma-Aldrich, Overijse, Belgium) diluted in PBS containing 1% bovine serum albumin (Sigma-Aldrich, Overijse, Belgium) (Table 2). After extensive rinsing with ice-cold PBS, samples were incubated with appropriate Alexa Fluor^®^ 594-conjugated secondary antibody (Jackson Immunoresearch Inc., West Grove, PA, USA) in blocking buffer. Nuclei were stained with DAPI and samples were mounted with Vectashield (Vector Laboratories, Burlingame, CA, USA). Detection was performed by fluorescence microscopy (40× objective) (Nikon Eclipse Ti, Tokyo, Japan). Images were processed and quantified with ImageJ software (version 1.52, USA). The ratio of the area of occurrence of the particular connexin to the number of nuclei was measured and expressed as relative alterations compared to untreated human hepatoma HepaRG cells, considered as control.

### 4.7. Statistical Analysis

Data were analyzed using GraphPad Prism 7 software and are presented as means +/− standard deviation (SD). The number of biological replicates (*n*) (i.e., in vivo experiments) or batches (*n*) (i.e., in vitro experiments) and technical replicates (N) (i.e., in vivo and in vitro experiments) are specified for each analysis in the figure legends. As such, 2-tailed Mann–Whitney tests or student t-tests with Welch’s correction were used to process the results of the analyses of the in vivo studies depending on the distribution (i.e., D’Agostino and Pearson normality test). A parametric one-way analysis of variance (ANOVA) followed by post hoc tests with Dunnett’s correction was used to process the results of the analyses of the in vitro experiments. Probability (*p*) values ≤ 0.05 were considered statistically significant.

## Figures and Tables

**Table 1:** Primers and probes used for RT-qPCR analysis of connexin and candidate reference genes. Assay identification (ID) for mouse (Mm; *Mus musculus*) or human (Hs; *Homo sapiens*) species, accession number, assay location, amplicon length and exon boundary of connexin and candidate reference genes are presented (*Gjb1*, Cx32; *Gjb2*, Cx26; *Gja1*, Cx43; *Actb*, β-actin; *B2m*, β-2-microglobulin; *Gapdh*, glyceraldehyde 3-phosphate dehydrogenase; *Hmbs*, hydroxymethylbilane synthase; *Ubc*, ubiquitin C).

Gene Symbol	Assay ID	Accession Number	Assay Location	Amplicon Size (Base Pairs)	Exon Boundary
*Gjb1*	*Mm*01950058_s1	NM_008124.2	466	65	1–1
*Gjb2*	*Mm*00433643_s1	NM_008125.3	603	72	2–2
*Gja1*	*Mm0*1179639_s1	NM_010288.3	2937	168	2–2
*Actb*	*Mm*00607939_s1	NM_007393.3	1233	115	6–6
*B2m*	*Mm*00437762_m1	NM_009735.3	111	77	1–2
*Gapdh*	*Mm*99999915_g1	NM_008084.2	265	107	2–3
*Hmbs*	*Mm*01143545_m1	NM_013551.2	473	81	6–7
*Ubc*	*Mm*02525934_g1	NM_019639.4	370	176	2–2
*Gjb1*	*Hs*00939759_s1	NM_000166.5	1547	63	2
*Gjb2*	*Hs*00269615_s1	NM_004004.5	715	123	2
*Gja1*	*Hs*00748445_s1	NM_000165.4	1031	142	2
*Actb*	*Hs*01060665_g1	NM_001101.3	208	63	2–3
*B2m*	*Hs*00187842_m1	NM_004048.2	134	64	1–2
*Gadph*	*Hs*02786624_g1	NM_001256799.2	870	157	7
*Hmbs*	*Hs*00609296_g1	NM_000190.3	1070	69	13–14
*Ubc*	*Hs*01871556_s1	M26880.1	2173	135	/

**Table 2:** Primary antibodies used for immunoblot (IB), immunohistochemistry (IHC) and immunocytochemistry analysis (ICC).

Antigen	Dilution			
	IB		IHC	ICC
	In Vitro	In Vivo		
Cx26	1/250	1/250	1/250	1/250
Cx32	1/600	1/1000	1/500	1/500
Cx43	1/1000	1/1000	1/100	1/1000
Nrf2	1/800	1/800	/	/

## Abbreviations

ANOVA	Analysis of variance
ATV	Atazanavir
BA(s)	Bile acid(s)
BDL	Bile duct ligation
Cx	Connexin
CsA	Cyclosporin A
DAPI	4′,6-diamidino-2-phenylindole
DIC	Drug-induced cholestasis
DILI	Drug-induced liver injury
DMSO	Dimethylsulfoxide
IB	Immunoblot
ICC	Immunocytochemistry
IHC	Immunohistochemistry
NEF	Nefazodone
Nrf2	Nuclear factor erythroid 2-related factor 2
PBS	Phosphate-buffered saline
*P*	Probability
RT-qPCR	Reverse transcription-quantitative polymerase chain reaction
SD	Standard deviation
